# Competition among Species of Stored-Product Psocids (Psocoptera) in Stored Grain

**DOI:** 10.1371/journal.pone.0102867

**Published:** 2014-08-08

**Authors:** Christos G. Athanassiou, Nickolas G. Kavallieratos, James E. Throne, Christos T. Nakas

**Affiliations:** 1 Laboratory of Entomology and Agricultural Zoology, Department of Agriculture, Crop Production and Rural Development, University of Thessaly, Nea Ionia Magnissia, Greece; 2 Laboratory of Agricultural Entomology, Department of Entomology and Agricultural Zoology, Benaki Phytopathological Institute, Kifissia, Attica, Greece; 3 USDA-ARS, Center for Grain and Animal Health Research, Manhattan, Kansas, United States of America; 4 USDA-ARS, San Joaquin Valley Agricultural Sciences Center, Parlier, California, United States of America; 5 Laboratory of Biometry, Department of Agriculture, Crop Production and Rural Development, University of Thessaly, Nea Ionia Magnissia, Greece; Federal University of Viçosa, Brazil

## Abstract

We evaluated the competition among stored-product psocid species by conducting two series of laboratory experiments. In the first series, three species of Liposcelididae were used: *Liposcelis bostrychophila*, *Liposcelis decolor*, and *Liposcelis paeta*. Five adult females of these species were placed in vials containing wheat, either alone or in all possible combinations of two species. The number of adults in the vials was counted after 35, 70, 105, 140, and 175 days. These tests were performed at 25 and 30°C. At 25°C, there were no differences in numbers of *L. bostrychophila* when this species was reared either alone or with each of the other two species. At 30°C, *L. bostrychophila* was the dominant species. The presence of *L. bostrychophila* had a negative effect on the growth of populations of *L. decolor* and *L. paeta*. The presence of *L. paeta* did not affect growth of populations of *L. decolor*, although the presence of *L. decolor* occasionally reduced growth of populations of *L. paeta*. In the second series of tests, *L. bostrychophila* adult females were placed in vials of wheat either alone or with adult females of *Lepinotus reticulatus*, at the ratios of (*L. bostrychophila*: *L. reticulatus*) 10∶0, 9∶1, 7∶3, 5∶5, 3∶7, 1∶9, and 0∶10. These tests were carried out only at 30°C, and the observation periods were the same as for the first series of tests. *Liposcelis bostrychophila* was the dominant species in this case as well, regardless of the ratio of the parental females. At the end of the experimental period, *L. reticulatus* was present only in vials that contained this species alone. Our results showed that *L. bostrychophila* outcompetes the other stored-product psocid species tested.

## Introduction

The insect community in the stored-grain agroecosystem consists of several categories of species, such as primary and secondary colonizers, fungus feeders, scavengers, predators, and parasitoids [1]. These species coexist and infest the product at various population densities [2], [3]. Hence, in contrast with field pests where pest control measures often can be directed toward control of one major pest species, pest control measures for durable stored products need to be selected to control the multiple species that coexist in these commodities. Despite this, the factors that determine species coexistence in durable stored products are poorly understood. During the last two decades only a few studies have examined the coexistence of multiple species and its possible incorporation into management practices. Nansen et al. [4], by examining large sample data sets from silos in Kansas, reported that the numbers of the red flour beetle, *Tribolium castaneum*, increased with the increase of the presence of the lesser grain borer, *Rhyzopertha dominica*, in the same sampling unit, but the numbers of these two species were negatively correlated with the presence of the rusty grain beetle, *Cryptolestes ferrugineus*. Athanassiou and Saitanis [5], in a flat wheat storage, found different spatiotemporal distribution between the Mediterranean flour moth, *Ephestia kuehniella*, and its parasitoids *Harbobracon hebetor* and *Venturia canescens*.

There are numerous laboratory studies investigating the competition among different stored-product insect species [6]–[11]. These studies were conducted at constant abiotic conditions, and the outcome of the competition was found to be highly affected by food availability [7], [12], life table characteristics of each species [9], [11], temperature and humidity [13], and the type of diet [14]. Theoretically, in studies where food availability is limited, it is generally expected that some species would be the “superior competitors” and that the “inferior competitors” are likely to become extinct [10], [15]. However, there were cases where the competitors were able to coexist for a long period, as long as food was available [8]. Nicholson [16] proposed two forms of intraspecific competition: contest and scramble competition. Contest competition was defined as the competition in which the winner species “obtains as much of the governing requisite as it needs for survival and reproduction” and the loser “relinquishes the requisite to its successful competitors”. Scramble competition was defined as the competition in which all the individuals of the population have equal access to the limited resource. These same two types of competition can also apply to interspecific competition.

Most of the studies that examine competition among stored-product insect species under laboratory conditions tested beetles, and, to a lesser extent, moths. There are no published data on competition among psocid (Psocoptera) pest species. Psocids, particularly some species of the family Liposcelididae, are major pests that can easily build up high populations, especially in stored grains [17]. They cause serious weight losses by consuming the germ and endosperm of grains, they contaminate commodities with feces and exuviae, they are a potential threat to human health through transfer of microorganisms, and they can cause allergic reactions in sensitized group of humans [18]–[24]. The elevated moisture content (>13%) of commodities allows microorganisms (e.g., fungi) to grow and consequently to affect their properties [25]–[29]. Psocid infestations are favored when the moisture content of commodities is high, and psocids can feed on fungi [30]. The coexistence of several psocid species in the same habitat can impact pest management because psocid species vary remarkably in their susceptibility to the insecticides commonly used for control of stored-product insect pests [31], [32]. Apart from their variable susceptibility to insecticides, psocid populations also develop at different rates on different types of food. In laboratory experiments with single species of psocids, Athanassiou et al. [33] showed that the type of food was one of the most important factors in population growth of four psocid species. Furthermore, abiotic factors also may have a substantial influence on developmental parameters [34]. Paradoxically, and despite the fact that species coexistence is very common in the case of stored-product psocids [35], [36], there are studies in which only one psocid species was primarily or even solely detected throughout long monitoring periods. For example, Opit et al. [37] found only *Liposcelis entomophila* in two bins of wheat in 2005, while in the same bins, they found only *L. decolor* one year later (2006) after fumigating the grain between years.

Despite the fact that coexistence of some major stored-product insect species has been investigated, there are no data available describing competition among species of stored-grain psocid pests. Thus, our study aims to provide the first series of data on the interspecific competition among species of stored-product psocid pests examining the long-term population growth of several psocid species placed alone (one species) or in pairs of species in the same food source.

## Materials and Methods

### Psocid rearing

*Liposcelis bostrychophila*, *L. decolor*, *L. paeta*, and *Lepinotus reticulatus* were used in the tests. *L. bostrychophila* and *L. reticulatus* are predominantly parthenogenetic, although a few males of *L. bostrychophila* were recently found [38]. All species were reared on a mixture of 97% cracked wheat kernels, 2% rice krispies, and 1% brewer's yeast at 30°C and 70% relative humidity, as suggested by Opit and Throne [34]. Adult females, <14-days old, were used in the tests.

### Experiment 1: Combinations of two species at constant densities

In these tests, we assessed each possible two-way combination of the three *Liposcelis* spp. (*L. bostrychophila* with *L. decolor*, *L. bostrychophila* with *L. paeta*, *L. decolor* with *L. paeta*), and also each species alone. For this purpose, 5 adult females of each species were placed in plastic vials (3 cm in diameter, 8 cm in height) with 5 g of untreated, clean, whole wheat (variety Fuller) adjusted to 13% moisture content. Five adult females were placed in vials where only one species was used. All vials then were placed in incubators set at 30°C and 75% relative humidity. The vials were opened after 35 days, and the adults of the two species were sieved from the wheat using a #30 sieve, identified, and counted. Nymphs were not identified nor counted, given that identification of nymphs of different species of the genus *Liposcelis* is difficult [39], and their inclusion in the counts might not have produced reliable results. This same procedure was repeated at 35-day intervals until 175 days from the start of the test (i.e., observation times were at 35, 70, 105, 140, and 175 days), using separate sets of vials for each observation time. The entire procedure was repeated at 25°C and 75% relative humidity. There were three sub-replicates, and the whole experiment was repeated 3 times by preparing new series of vials each time. Thus, 9 vials (3 replicates×3 sub-replicates) in total were prepared for each insect combination-temperature-observation for a total of 154 vials (9 vials×5 observations periods×2 temperatures×6 species combinations) altogether for the entire experiment.

### Experiment 2: Combinations of two parthenogenetic species at various densities

In these tests, *L. bostrychophila* and *L. reticulatus* were used. Ten adult females were placed in each vial at the following combinations of *L. bostrychophila*: *L. reticulatus* adult ratios: 10∶0, 9∶1, 7∶3, 5∶5, 3∶7, 1∶9, and 0∶10. The experimental procedure, the conditions, and the observation periods were as described above, with the exception that this experiment was carried out only at 30°C. In these tests, both adults and nymphs were identified and counted, given that all stages of the genera *Liposcelis* and *Lepinotus* can be distinguished easily [40], [41]. There were three replicates with three sub-replicates each time (9 vials for each case) for a total of 315 vials (9 vials×5 observation periods×7 species combination ratios).

### Data analysis

For Experiment 1, the data were analyzed, separately for each species, using a three-way ANOVA with temperature, observation period, and psocid species as main effects, with psocid counts as the response variable. Similarly, for Experiment 2, the data were analyzed using a two-way ANOVA, with observation period and species ratio as main effects. All analyses were conducted using the JMP 10 software (SAS Institute Inc., Cary, NC, U.S.A.). Means were separated by the use of the Tukey-Kramer HSD test at α = 0.05 [42].

The evolution of adult counts for the different insect species was modelled as a function of time using a quadratic regression model with temperature and initial counts of insects as covariates. The quadratic term for time was preferable to alternatives given the fact that it allows the possibility of capturing U-shaped patterns and steep rates of decline as time increases [43]. This strategy was also used for modelling the evolution of nymph counts for the different insect species. Suitability of models was determined by examination of residuals and R^2^. All analyses were performed using R version 3.0.2 (R Project for Statistical Computing, http://www.r-project.org)

## Results

### Experiment 1

For *L. bostrychophila*, all main effects were significant, but only the Period×Temperature interaction was significant (Table 1). At 25°C, there were no significant differences in the numbers of *L. bostrychophila* among the three species combinations at any observation time (Table 2). At 30°C, differences among species combinations were observed only after 70 and 105 days. After 70 days at 30°C, there were more *L. bostrychophila* when reared alone than when they were reared with *L. decolor*, while after 105 days where there were more *L. bostrychophila* when reared alone than when they were reared with *L. paeta*. The general pattern was that there were numerically more *L. bostrychophila* when reared alone at 30°C than when they were reared with another species. At 25°C, populations exceeded 300 psocids in all treatments at their peak, while, at 30°C, this population level was approached only when *L. bostrychophila* were reared alone and peak population levels were about half that in the other two treatments. Populations at both temperatures began to decrease during the course of the study, with this occurring at 175 days at 25°C and generally at 140 days at 30°C.

**Table 1:** ANOVA parameters for main effects and interactions for the three psocid species tested.

		Psocid species					
		*L. bostrychophila*		*L. decolor*		*L. paeta*	
Source	df	F	p	F	p	F	p
Period	4	50.7	<0.01	14.5	<0.01	2.6	0.04
Temperature	1	75.8	<0.01	33.3	<0.01	182.3	<0.01
Species	2	8.2	<0.01	40.2	<0.01	69.5	<0.01
Period×Temperature	4	48.4	<0.01	4.8	<0.01	6.4	<0.01
Period×Species	8	1.4	0.20	3.1	<0.01	1.1	0.34
Temperature×Species	2	3.0	0.05	5.5	<0.01	46.5	<0.01
Period×Temperature×Species	8	1.2	0.32	1.8	0.08	2.6	<0.01

In all cases, total df = 269.

**Table 2:** Mean number of *L. bostrychophila* adults ± SE per vial (for vials in which the initial population was 5 adult females of *L. bostrychophila* alone, or 5 adult females of *L. bostrychophila* with 5 adult females of *L. decolor*, or 5 adult females of *L. bostrychophila* with 5 adult females of *L. paeta*) at 35, 70, 105, 140, and 175 days after the introduction of the initial population at two temperatures.

	Period (days after introduction of the first females)				
Initial species in the vial	35	70	105	140	175
	25°C				
*L. bostrychophila*	28.0±2.7	121.8±7.1	256.9±29.1	344.7±37.0	266.4±28.2
*L. bostrychophila* with *L. decolor*	21.7±3.0	106.2±10.3	231.3±35.5	341.6±41.2	211.5±35.8
*L. bostrychophila* with *L. paeta*	25.9±3.3	118.4±18.2	240.7±19.5	320.8±33.0	258.6±23.2
F	1.1	0.4	0.2	0.1	1.0
p	0.34	0.66	0.82	0.89	0.38
	30°C				
*L. bostrychophila*	65.1±14.2	253.2±30.0 a	278.7±32.7 a	157.7±46.8	18.6±8.9
*L. bostrychophila* with *L. decolor*	46.2±6.0	146.2±25.4 b	166.2±39.5 ab	92.9±32.8	14.6±6.9
*L. bostrychophila* with *L. paeta*	54.8±6.8	188.6±16.1 ab	114.9±31.1 b	64.7±10.6	30.5±9.0
F	1.0	4.8	5.8	2.0	0.99
p	0.40	0.02	<0.01	0.15	0.39

Within each column and temperature, means followed by the same letter are not significantly different; where no letters exist, no significant differences were noted (Tukey-Kramer HSD test at p = 0.05; in all cases, df = 2, 24).

For *L. decolor*, all main effects and interactions were significant, with the exception of Period×Temperature×Species (Table 1). At 25°C and at all observation times, significantly more *L. decolor* adults were found in vials containing either *L. decolor* alone or *L. decolor* with *L. paeta* than in vials containing *L. decolor* with *L. bostrychophila* (Table 3). The same pattern was observed at 30°C after 35 days, but there were no differences among the species combination treatments at later observation times. The *L. decolor* population died out by 140 and 105 days when reared with *L. bostrychophila* at 25 and 30°C, respectively. *L. decolor* populations never reached 30 individuals at 25°C in any treatment and never reached 20 individuals at 30°C. Only a few *L. decolor* adults were found in any of the species combination treatments after 175 days at 30°C, and only in vials where this species was reared alone.

**Table 3:** Mean number of *L. decolor* adults ± SE per vial (for vials in which the initial population was 5 adult females of *L. decolor* alone, or 5 adult females of *L. decolor* with 5 adult females of *L. bostrychophila*, or 5 adult females of *L. decolor* with 5 adult females of *L. paeta*) at 35, 70, 105, 140, and 175 days after the introduction of the initial population at two temperatures.

	Period (days after introduction of the first females)				
Initial species in the vial	35	70	105	140	175
	25°C				
*L. decolor*	13.1±3.4 a	19.6±10.3 a	29.9±7.3 a	6.9±2.7 a	7.6±2.9 a
*L. decolor* with *L. bostrychophila*	5.0±1.2 b	4.1±1.0 b	0.6±0.4 b	0.0±0.0 b	0.0±0.0 b
*L. decolor* with *L. paeta*	13.0±1.6 a	20.4±3.0 a	18.7±3.8 a	9.2±1.8 a	6.9±2.0 a
F	4.4	6.4	9.6	6.6	4.2
p	0.03	<0.01	<0.01	<0.01	0.03
	30°C				
*L. decolor*	18.6±4.6 a	10.0±4.6	7.7±3.5	2.2±1.1	0.2±0.1
*L. decolor* with *L. bostrychophila*	3.8±0.9 b	0.4±0.2	0.0±0.0	0.1±0.1	0.0±0.0
*L. decolor* with *L. paeta*	9.9±1.1 ab	10.6±2.9	4.1±1.1	2.4±1.5	0.0±0.0
F	7.3	3.3	3.3	1.3	2.3
p	<0.01	0.06	0.06	0.28	0.12

Within each column and temperature, means followed by the same letter are not significantly different; where no letters exist, no significant differences were noted (Tukey-Kramer HSD test at p = 0.05; in all cases, df = 2, 24).

For *L. paeta*, all main effects and interactions were significant, with the exception of Period×Species (Table 1). At 25°C at all observation times other than the first, there were generally fewer *L. paeta* when reared with *L. bostrychophila* than when *L. paeta* was reared alone (Table 4). *L. paeta* population levels did not differ significantly whether reared with *L. decolor* or *L. bostrychophila*. *L. paeta* population levels did not differ significantly when reared alone or with *L. decolor*, except after 175 days. At 30°C for the first 105 days, there were more *L. paeta* when they were reared alone than when they were reared with *L. decolor*, and the fewest *L. paeta* were found when they were reared with *L. bostrychophila*. After 105 days at 30°C, the numbers of *L. paeta* did not differ when they were reared alone or with *L. decolor*, but fewer were found when *L. paeta* was reared with *L. bostrychophila*. The number of *L. paeta* adults was considerably higher at 30 than at 25°C (Table 4). At 25°C, the number of adults found never exceeded 10 individuals, except after 175 days when *L. paeta* was reared alone. At 30°C, a maximum of 69 *L. paeta* were found after 70 days when they were reared alone, 37 after 70 days when reared with *L. decolor*, and only 9 at the first observation time when reared with *L. bostrychophila*.

**Table 4:** Mean number of *L. paeta* adults ± SE per vial (for vials in which the initial population was 5 adult females of *L. paeta* alone, 5 adult females of *L. paeta* with 5 adult females of *L. bostrychophila*, or 5 adult females of *L. paeta* with 5 adult females of *L. decolor*) at 35, 70, 105, 140, and 175 days after the introduction of the initial population at two temperatures.

	Period (days after introduction of the first females)				
Initial species in the vial	35	70	105	140	175
	25°C				
*L. paeta*	4.0±0.6	7.3±1.5 a	5.2±0.5 a	4.1±1.4	19.2±3.7 a
*L. paeta* with *L. bostrychophila*	4.4±1.2	2.9±0.9 b	3.1±0.5 b	2.0±0.7	3.7±0.8 b
*L. paeta* with *L. decolor*	3.8±0.7	3.4±0.9 ab	3.2±0.7 ab	3.4±0.6	7.1±2.5 b
F	0.1	4.4	3.7	0.2	9.1
p	0.87	0.02	0.04	0.79	<0.01
	30°C				
***L. paeta***	48.3±2.5 a	69.4±10.8 a	48.4±7.6 a	43.0±6.3 a	24.9±7.6 a
*L. paeta* with *L. bostrychophila*	8.7±2.7 c	6.6±1.0 c	2.6±1.1 c	2.4±1.1 b	2.7±1.1 b
*L. paeta* with *L. decolor*	28.9±6.5 b	37.1±7.4 b	27.1±6.7 b	31.3±10.1 a	25.5±5.2 a
F	20.9	17.1	15.2	9.1	6.0
p	<0.01	<0.01	<0.01	<0.01	<0.01

Within each column and temperature, means followed by the same letter are not significantly different; where no letters exist, no significant differences were noted (Tukey-Kramer HSD test at p = 0.05; in all cases, df = 2, 24).

**Figure 1:**
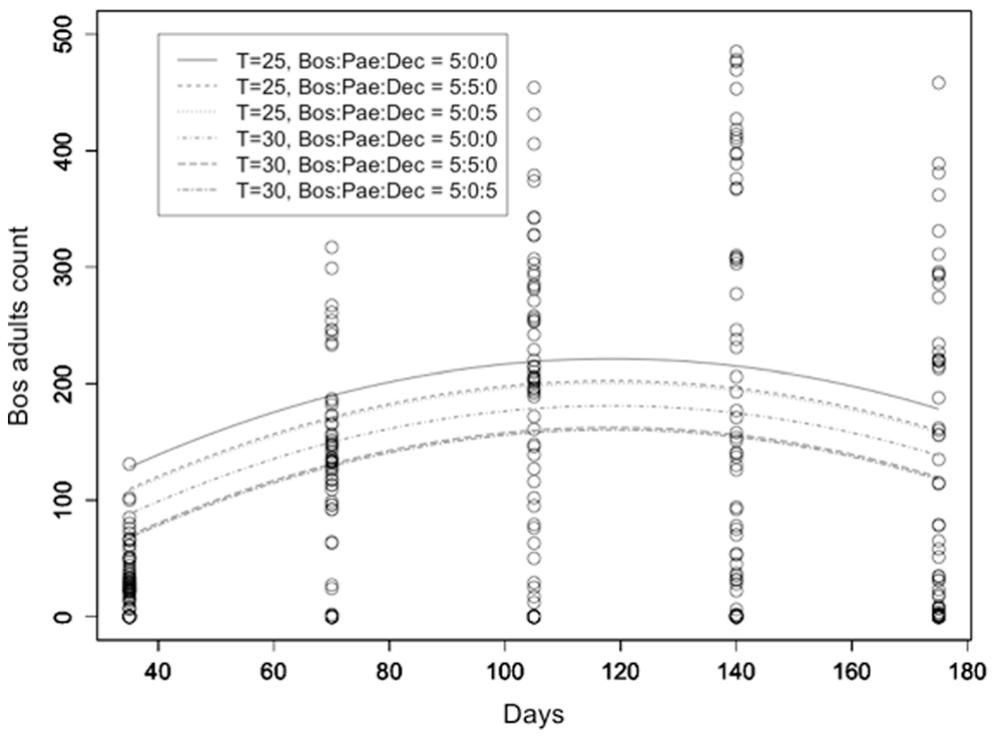
*L. bostrychophila* adult counts. *L. bostrychophila* adult counts at 25 and 30°C as a function of time along with the fitted models under different competition scenarios (Bos = *L. bostrychophila*, Pae = *L. paeta*, Dec = *L. decolor*).

The population growth (population evolution) of *L. bostrychophila* adult counts as a function of time (in days) was modeled using a quadratic regression model taking into account the *L. bostrychophila*: *L. paeta*: *L. decolor* initial counts and temperature (R^2^ = 0.535; Fig. 1). All predictors had a significant effect (p<0.05). Specifically, there was a significant evolution of *L. bostrychophila* adults counts during time (t = 8.719, p<0.001 for the linear and t = −7.881, p<0.001 for the quadratic term). The initial number of *L. paeta* had a significant effect on *L. bostrychophila* adults counts (t = −2.193, p = 0.029) and similarly for *L. decolor* (t = −2.422, p = 0.016). The effect of temperature was highly significant with lower temperature resulting in higher adult *L. bostrychophila* counts (t = −5.763, p<0.001).

**Figure 2:**
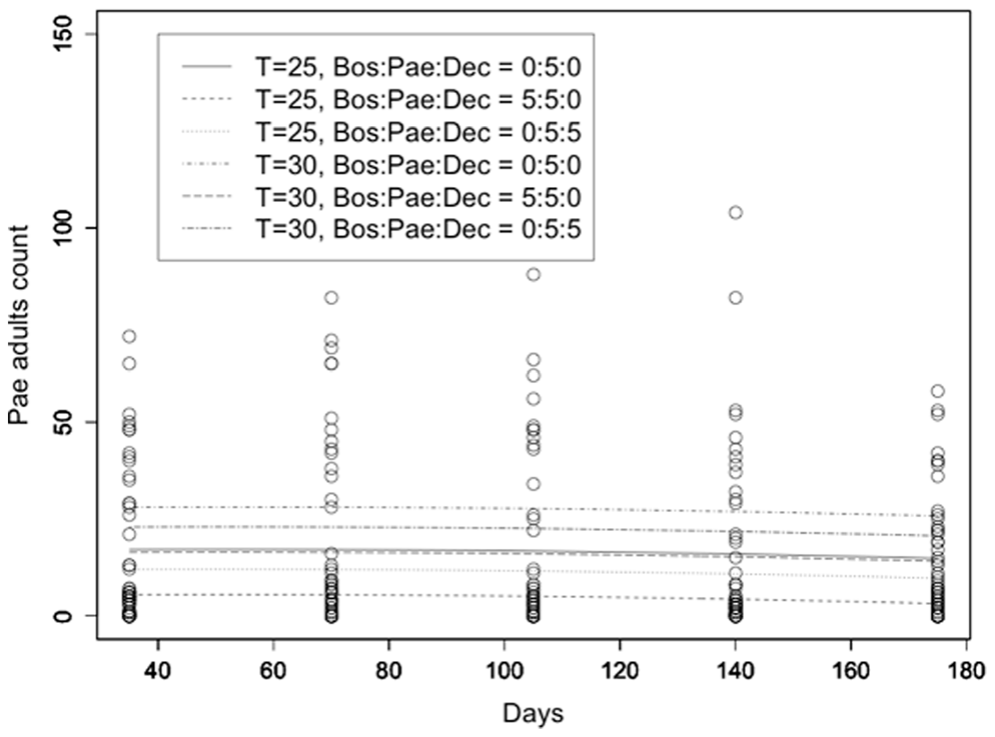
*L. paeta* adult counts. *L. paeta* adult counts at 25 and 30°C as a function of time along with the fitted models under different competion scenarios (Bos = *L. bostrychophila*, Pae = *L. paeta*, Dec = *L. decolor*).

The population evolution of *L. paeta* adult counts as a function of time (in days) was modeled as for *L. bostrychophila* (R^2^ = 0.394; Fig. 2). *L. paeta* adult counts did not significantly change over time, while all other predictors had a significant effect (p<0.001). There was no significant evolution of *L. paeta* adult counts over time (t = 0.251, p = 0.802 for the linear and t = −0.530, p = 0.596 for the quadratic term). However, the initial number of *L. bostrychophila* had a significant effect on *L. paeta* adult counts (t = −8.132, p<0.001), while the same was found for *L. decolor* (t = −3.583, p<0.001). The effect of temperature was highly significant with lower temperature resulting in lower adult *L. paeta* counts (t = 9.338, p<0.001).

**Figure 3:**
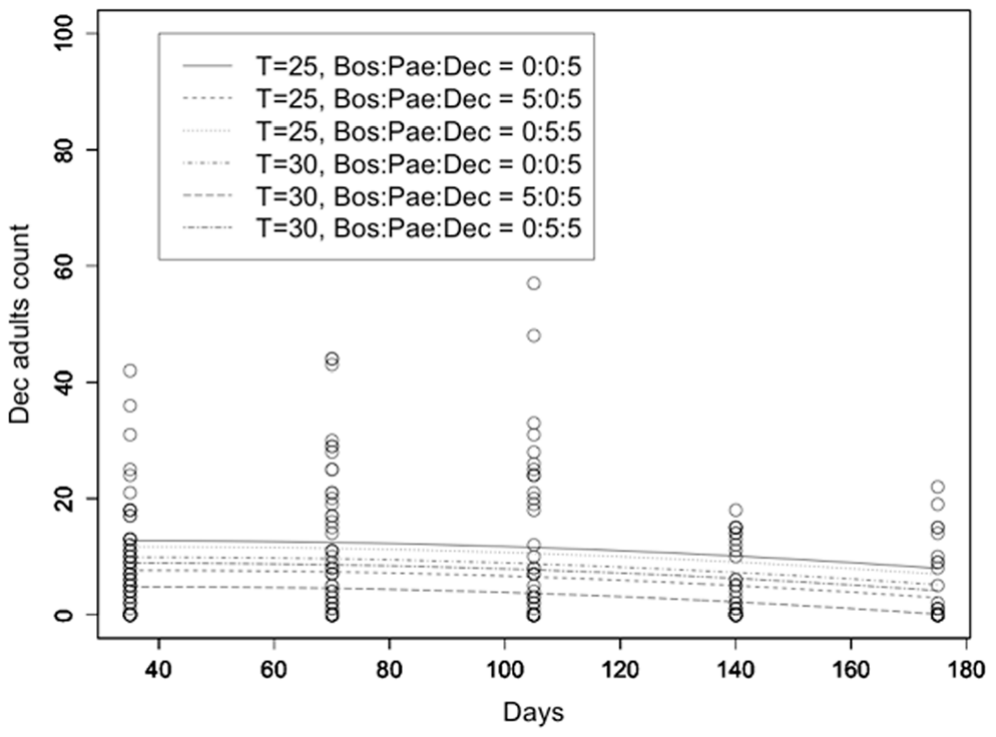
*L. decolor* adult counts. *L. decolor* adult counts at 25 and 30°C as a function of time along with the fitted models under different competition scenarios (Bos = *L. bostrychophila*, Pae = *L. paeta*, Dec = *L. decolor*).

Finally, the population of *L. decolor* adult counts was similarly modeled (R^2^ = 0.333; Fig. 3). *L. decolor* adult counts did not significantly change over time and were independent of the initial number of *L. paeta*. Predictors such as temperature and initial number of *L. bostrychophila* had a significant effect (p<0.001). There was no significant evolution of *L. decolor* adults' counts over time (t = 0.603, p = 0.547 for the linear and t = −1.714, p = 0.087 for the quadratic term). The initial number of *L. bostrychophila* had a significant effect on *L. decolor* adult counts (t = −6.935, p<0.001). However, the initial number of *L. paeta* did not significantly affect *L. decolor* counts (t = −1.401, p = 0.162). The effect of temperature was highly significant with lower temperature resulting in higher adult *L. decolor* counts (t = −4.717, p<0.001).

### Experiment 2

For *L. bostrychophila* adults and nymphs, Period and Species ratio×Period were significant, but Species ratio was not (Table 5). After 35 and 70 days, the number of *L. bostrychophila* adults was significantly greater as the initial number of *L. bostrychophila* adults in the vial increased (Table 6), with numbers of adults ranging from 3 to 24 and 119 to 168 after 35 and 70 days, respectively. Numbers of nymphs varied with initial density only after 35 days. After 105 and 140 days, there were no significant differences among treatments in the numbers of nymphs or adults. In contrast, after 175 days, the number of adults tended to decrease as the initial number of parental females increased. In fact, greater than 2 times more adults were found in vials that initially contained 1 parental *L. bostrychophila* female than in vials that initially contained 10 parental *L. bostrychophila* females.

**Table 5:** ANOVA parameters for main effects and their interaction for the two psocid species tested.

		Species/life stage											
		*L. bostrychophila*						*L. reticulatus*					
		adults		nymphs		total		adults		nymphs		Total	
Source	df	F	p	F	p	F	p	F	p	F	p	F	p
Species ratio	5	66.7	0.65	0.3	0.92	0.4	0.85	50.2	<0.01	26.0	<0.01	57.0	<0.01
Period	4	66.7	<0.01	24.0	<0.01	316.7	<0.01	12.5	<0.01	50.0	<0.01	13.5	<0.01
Species ratio×Period	20	2.5	<0.01	4.3	<0.01	2.3	<0.01	3.4	<0.01	2.0	<0.01	1.7	0.03

In all cases, total df = 269.

**Table 6:** Mean number of adults, nymphs, or total number of adults and nymphs of *L. bostrychophila* ± SE per vial (for vials in which the initial population was adult females of *L. bostrychophila* with adult females of *L. reticulatus* at a ratio of 1∶9, 3∶7, 5∶5, 7∶3, 9∶1, and 10∶0 adults (*L. bostrychophila*: *L. reticulatus*)) at 35, 70, 105, 140, and 175 days after the introduction of the initial population.

	Period (days after introduction of the first females)				
Adult ratio	35	70	105	140	175
	Adults				
1∶9	3.4±1.3 c	19.2±5.5 d	165.1±29.5	209.3±44.1	313.6±49.9 a
3∶7	9.2±1.6 bc	58.4±7.7 c	185.1±19.3	200.7±32.3	277.2±38.0 ab
5∶5	11.7±1.6 bc	86.2±9.7 c	182.1±32.1	226.3±40.1	183.6±29.4 ab
7∶3	14.3±1.3 b	93.1±11.0 bc	236.9±30.7	238.4±47.7	217.1±34.9 ab
9∶1	18.5±2.8 ab	139.2±14.6 ab	219.0±25.7	233.0±40.6	208.6±37.3 ab
10∶0	24.3±3.8 a	168.0±18.1 a	209.0±16.5	200.9±32.0	134.7±31.3 b
F	10.4	20.5	1.0	0.2	3.0
p	<0.01	<0.01	0.41	0.97	0.02
	Nymphs				
1∶9	14.7±2.7 d	50.7±15.0	94.6±16.7	43.4±14.8	34.4±12.6
3∶7	34.0±3.0 c	69.6±6.3	86.8±22.9	33.8±11.3	19.7±4.1
5∶5	34.0±8.6 bc	76.2±15.0	90.9±22.3	33.3±11.0	19.6±5.5
7∶3	74.1±5.2 b	44.4±10.2	91.1±29.7	22.2±6.4	20.2±7.1
9∶1	96.7±8.5 a	59.9±13.6	61.0±17.7	26.1±12.2	15.1±5.4
10∶0	141.4±9.6 a	50.8±11.9	42.4±14.3	17.1±3.3	8.1±3.2
F	43.4	0.9	0.9	0.8	1.6
p	<0.01	0.43	0.43	0.50	0.17
	Total				
1∶9	18.1±3.5d	69.9±20.0c	259.7±43.0	254.8±56.5	349.0±60.5 a
3∶7	43.2±3.2c	128.0±11.9bc	271.9±33.4	234.4±38.3	296.9±41.1 ab
5∶5	75.4±9.7bc	162.4±20.9ab	273.0±49.4	259.7±49.0	203.1±33.6 ab
7∶3	88.4±6.2b	137.6±14.2bc	328.0 ± 52.8	260.7±52.4	237.3±33.6 ab
9∶1	115.8±8.8a	199.1±20.2ab	280.0±40.4	259.1±50.6	223.7±41.6 ab
10∶0	165.8±9.5a	218.7±17.3a	251.4±16.8	218.6±34.3	142.8±32.0 b
F	51.2	9.1	0.4	0.1	3.0
p	<0.01	<0.01	0.82	0.98	0.02

Within each column and life stage, means followed by the same letter are not significantly different; where no letters exist, no significant differences were noted; HSD test at *P* = 0.05; in all cases, df = 5, 53).

For *L. reticulatus*, all main effects and their interaction were significant (Table 5). Throughout the study, both the numbers of *L. reticulatus* nymphs and adults increased as the initial number of *L. reticulatus* parental females in the vials increased (Table 7). A maximum of only 35 *L. reticulatus* adults or 33 nymphs was produced during the study and only when this species was reared alone, and the initial number of adults was exceeded in only a few cases. After 175 days, *L. reticulatus* had gone extinct except in vials where they were reared alone.

**Table 7:** Mean number of adults, nymphs, or total number of adults and nymphs of *L. reticulatus* ± SE per vial (for vials in which the initial population was females of *L. reticulatus* with adult females of *L. bostrychophila* at a ratio of 1∶9, 3∶7, 5∶5, 7∶3, 9∶1, and 10∶0 adults (*L. reticulatus*: *L. bostrychophila*)) at 35, 70, 105, 140, and 175 days after the introduction of the initial population.

	Period (days after introduction of the first females)				
Adult ratio	35	70	105	140	175
	Adults				
1∶9	0.6±0.3 c	2.7±1.7 c	1.6±1.1 b	0.0±0.0 b	0.0±0.0 b
3∶7	1.7±0.4 bc	2.4±0.8 c	4.6±2.7 b	0.0±0.0 b	0.0±0.0 b
5∶5	2.2±0.8 bc	9.2±2.7 bc	4.4±2.6 b	0.0±0.0 b	0.0±0.0 b
7∶ 3	2.9±0.8 bc	11.4±2.9 bc	3.1±2.0 b	0.0±0.0 b	0.0±0.0 b
9∶1	5.7±1.3 ab	20.8±4.3 ab	5.2±3.6 b	1.9±1.9 b	0.0±0.0 b
10∶0	8.2±2.2 a	31.9±6.9 a	35.3±6.3 a	33.7±8.2 a	17.4±4.1 a
F	5.9	9.0	14.0	15.7	17.7
p	<0.01	<0.01	<0.01	<0.01	<0.01
	Nymphs				
1∶9	5.4±1.9 c	0.6±0.4 b	0.0±0.0 b	0.0±0.0 b	0.0±0.0 b
3∶7	10.2±2.0 bc	1.6±0.7 b	0.0±0.0 b	0.0±0.0 b	0.0±0.0 b
5∶5	14.3±2.7 bc	2.4±0.8 ab	0.0±0.0 b	0.0±0.0 b	0.0±0.0 b
7∶3	16.8±3.6 bc	2.2±1.2 ab	0.2±0.2 b	0.0±0.0 b	0.0±0.0 b
9∶1	24.1±4.2 ab	5.2±2.0 ab	0.2±0.1 b	0.2±0.2 b	0.0±0.0 b
10∶0	33.1±6.6 a	10.3±4.0 a	8.3±2.6 a	14.2±4.9 a	12.6±4.4 a
F	6.7	3.4	9.9	8.4	8.2
p	<0.01	0.01	<0.01	<0.01	<0.01
	Total				
1∶ 9	6.0±2.2 c	3.2±2.2 c	1.5±1.1 b	0.0±0.0 b	0.0±0.0 b
3∶ 7	11.9±2.3 bc	4.0±1.2 c	4.5±2.7 b	0.0±0.0 b	0.0±0.0 b
5∶ 5	16.6±3.1 bc	11.7±3.1 bc	4.4±2.6 b	0.0±0.0 b	0.0±0.0 b
7∶ 3	19.7±4.2 bc	13.6±4.0 bc	3.3±2.2 b	0.0±0.0 b	0.0±0.0 b
9∶ 1	27.7±5.3 ab	26.0±5.3 ab	5.4±3.7 b	2.1±2.1 b	0.0±0.0 b
10∶ 0	41.3±8.2 a	42.2±9.9 a	43.7±7.0 a	47.9±10.7 a	30.0±11.9 a
F	7.4	8.5	19.5	19.1	14.6
p	<0.01	<0.01	<0.01	<0.01	<0.01

Within each column and life stage, means followed by the same letter are not significantly different; where no letters exist, no significant differences were noted; HSD test at *P* = 0.05; in all cases, df = 5, 53).

**Figure 4:**
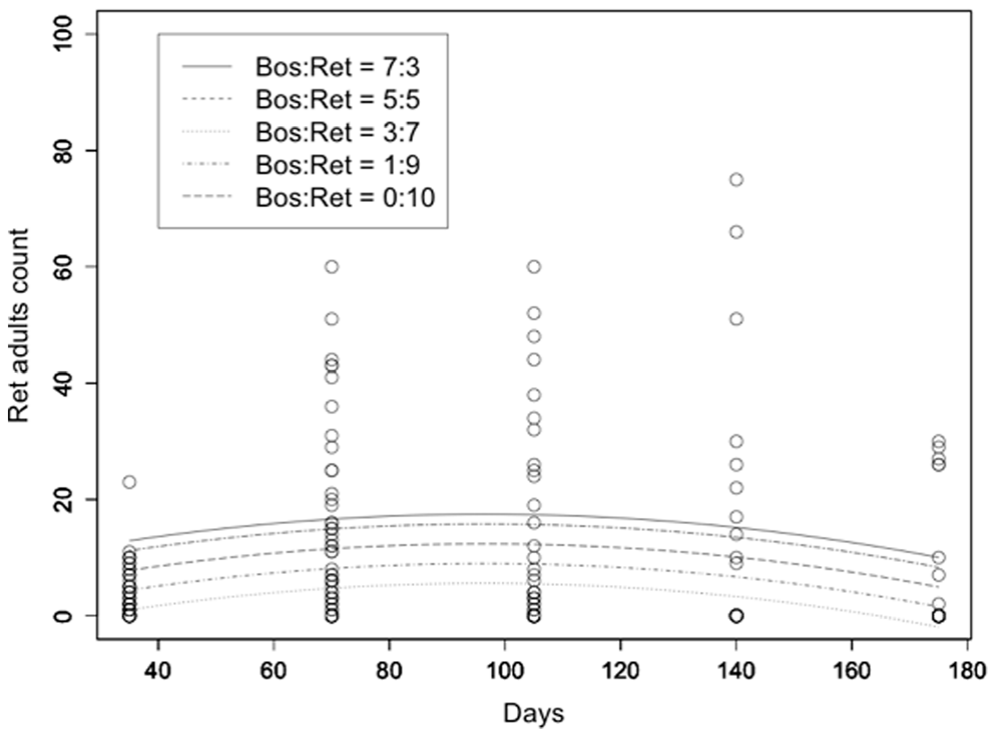
*L. reticulatus* adult counts. *L. reticulatus* adult counts as a function of time along with model fit under different competition scenarios (Bos = *L. bostrychophila*, Ret = *L. reticulatus*).

**Figure 5:**
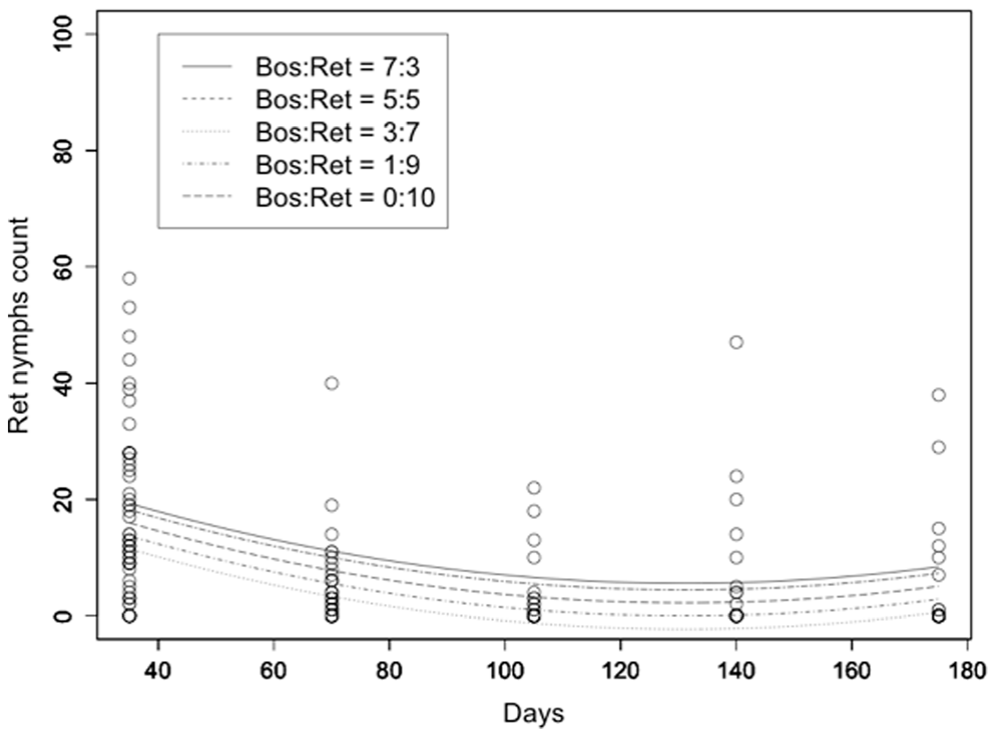
*L. reticulatus* nymph counts. *L. reticulatus* nymph counts as a function of time along with model fit under different competition scenarios (Bos = *L. bostrychophila*, Ret = *L. reticulatus*).

**Figure 6:**
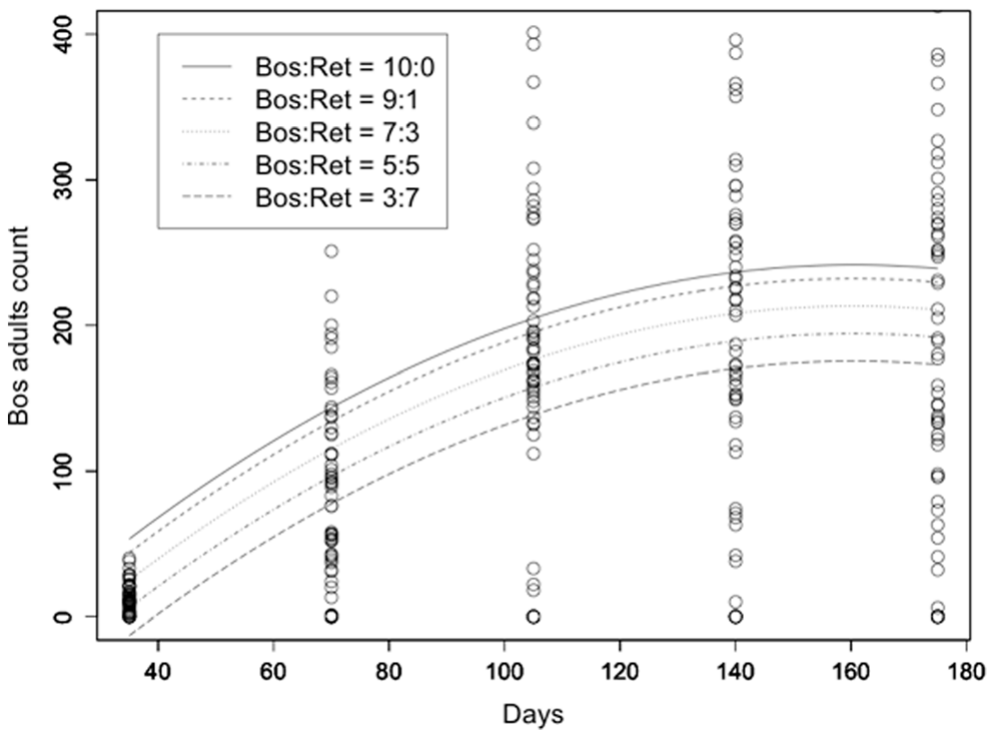
*L. bostrychophila* adult counts. *L. bostrychophila* adult counts as a function of time along with model fit under different competition scenarios (Bos = *L. bostrychophila*, Ret = *L. reticulatus*).

**Figure 7:**
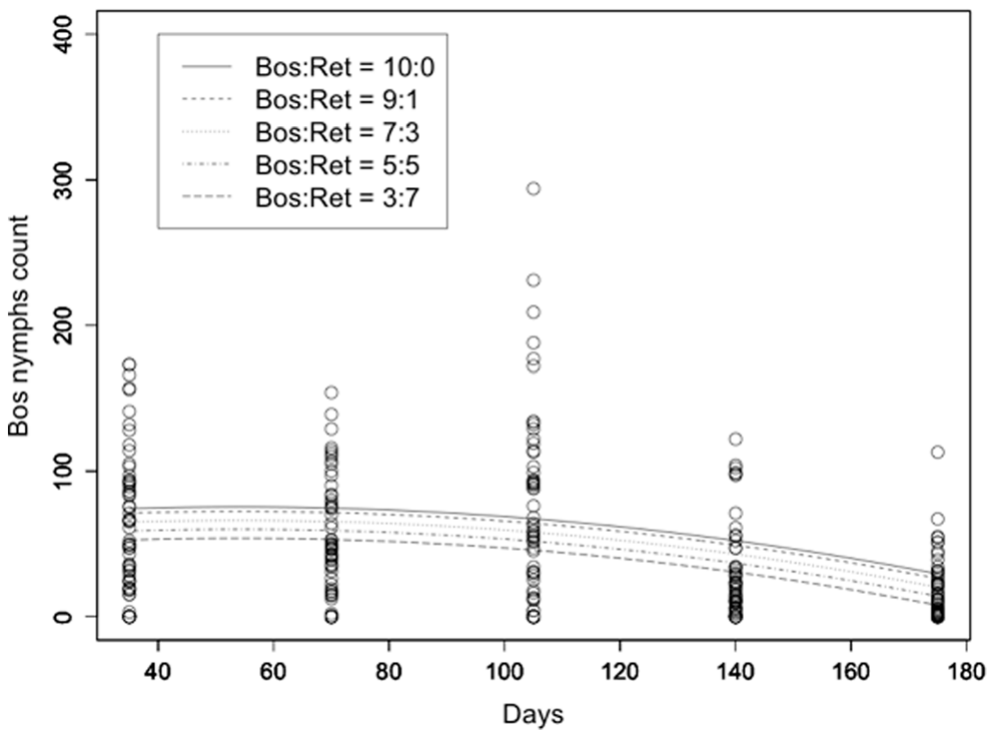
*L. bostrychophila* nymph counts. *L. bostrychophila* nymph counts as a function of time along with model fit under different competition scenarios (Bos = *L. bostrychophila*, Ret = *L. reticulatus*).

The model used for the description of the population evolution of *L. reticulatus* adult counts over time included only the linear and quadratic terms for time and the initial *L. reticulatus*: *L. bostrychophila* rate. All terms were highly statistically significant with t>3.5 and p<0.001. R^2^ was equal to 0.289 (Fig. 4). Similar results were obtained for the evolution of *L. reticulatus* nymph counts with R^2^ = 0.412 (Fig. 5). Respective results for *L. bostrychophila* are shown in Figs. 6, and 7. R^2^ for *L. bostrychophila* adult counts was 0.404 and for *L. bostrychophila* nymph counts 0.183.

## Discussion

The results of the present study document that psocid species can coexist for a relatively long interval, but this coexistence is directly affected by the species that share the same food source. In the series of tests with the three *Liposcelis* species, our tests showed that *L. decolor* and *L. paeta* can coexist; hence, scramble competition seems to be the dominant competition type for these two species. Nevertheless, for both species, the simultaneous presence of *L. bostrychophila* negatively affected their population growth, regardless of the temperature level. In fact, coexistence with *L. bostrychophila* caused extinction of *L. decolor* by the end of the study, and the number of *L. decolor* adults was reduced, even after the first 35 days of observation. Despite the fact that *L. paeta* populations did not go extinct, the presence of *L. bostrychophila* drastically reduced its population growth in comparison with either *L. paeta* alone, or *L. paeta* with *L. decolor*. In contrast, *L. bostrychophila* population growth was not affected by the presence of either of the other species. Consequently, among the species tested, *L. bostrychophila* has the ability to be the “superior colonizer”, a species that can easily build up high populations in relatively short periods, and remain unaffected by the presence of other species, at least under the conditions tested here. Several studies documented that *L. bostrychophila* is one of the most common psocid species in stored grains and related commodities [23], [44]–[47]. Our results show that the rapid population growth of *L. bostrychophila* could be partially attributed to its ability to overcome competition with other major *Liposcelis* species.

Long-term laboratory studies on the competition of stored-product insects have been based on protocols in which the medium is replaced to ensure continuous food availability [8], [9], [11]. Conversely, the basic characteristic of the current study was that the entire experiment was carried out in a specific quantity of the commodity, which should be regarded as the decisive factor that defined the outcome of the competition at the end of the experimental period, but also regulated the interspecific competition trends. In cases of sufficient food availability, several species may coexist for a long interval by modifying their spatiotemporal distributions to local foci of infestation [3]. However, limited availability of food is directly related to individual (per capita) vital rates, which lead to environmentally-mediated density dependent characteristics [8],[48],[49]. During the last observation period of the experiment (175 days), we noticed that there was no food available in the vials or in some cases the food available in the vials was negligible due to the continuous infestation and the resulting degradation. Food exhaustion seriously moderates reproduction and survival. In our study, we observed that, even for *L. bostrychophila* which had the highest numbers in both tests, populations started to decline after a peak, which usually occurred during the 105 and/or 140 days observation period. Moreover, this trend also was observed even when only one species was present in the vials, which clearly indicates density-dependent regulating mechanisms [11], [49]. It is generally considered that population growth has a certain cost in fecundity rates. For example, Giga and Smith [8] reported that fecundity of the cowpea weevil, *Callosobruchus maculatus*, was negatively affected by an increase in adult density. Still, Lale and Vidal [11] reported that this species had the ability to develop easily under conditions of high population density. It should be noted that the adults of this species may use semiochemicals to mark the seeds infested [50]. According to Cameron et al. [15], the patterns of density-dependent resource competition are expressed more vigorously under resource limitations.

Kučerova [23] found that *L. bostrychophila* was able to cause approx. 10% weight loss in broken wheat kernels after 3 months, and that during this interval populations increased more than 100 times. Our results are in accordance with that observation. This clearly implies that some stored-product psocids are able to develop, probably at different food uptake rates, in waste products, which allow growth at very low levels of food availability. For both tests, populations decreased at the end of the observation period, so we assume that as food availability becomes limited, severe intraspecific resource competition bottlenecks regulate population growth [51].

In a recent study, Athanassiou et al. [33] found that *L. bostrychophila* was able to develop higher numbers than *L. decolor* and *L. paeta* in various types of grain commodities, especially when cracked kernels were present. *L. decolor* had the lowest rate of population growth regardless of the commodity. This stands in accordance with the results of the present study, where *L. decolor* had the lowest number of adults among the three *Liposcelis* species tested, even in vials where it was the only species. Hence, population growth trends of this species may be an indirect outcome of its slower developmental rates on this commodity or varieties of this commodity so far used for experiments, rather than an outcome of competition with other species. On an optimal commodity, *L. decolor* development rates are quicker than for *L. bostrychophila* or *L. paeta* at 25 and 30°C [17].

As expected, temperature played an important role in population growth and the concomitant competition. For *L. bostrychophila*, there was no effect of the presence of other species at 25°C, throughout the entire experimental period. In contrast, at 30°C, during the observation periods when psocid populations were high (70–140 days), *L. bostrychophila* population levels were lower when other species were present. The analysis performed here suggested that despite the fact that this species was always the winner of the competition, its population growth was highly mediated by the presence of other species, which means that competition is likely to slow population increase in a more realistic scenario of a larger spatial scale. This clearly indicates that competition, at least for *L. bostrychophila*, is highly mediated by temperature, and, at low temperatures, coexistence may occur. In contrast, the adult population of *L. decolor* was continuously higher when this species was reared alone at 25°C than when this species coexisted with *L. bostrychophila*. Moreover, there were no differences in *L. decolor* adult numbers at 30°C for the last 140 days of the observation period. *L. paeta* adult numbers were reduced at both temperatures when reared with other species. Rees and Walker [52] found that at 30°C, population growth of *L. bostrychophila* was three times higher than that of *L. paeta*, but the differences were minimal at lower temperatures. Finally, for both temperature levels tested here, egg-to-adult survivorship was higher for *L. bostrychophila* than for *L. decolor* or *L. paeta* [53]–[55]. These studies may partially explain our results for the effect of temperature on the competition of different species. Our results clearly indicate that reproduction was rapid for *L. bostrychophila* at 25°C during the first 35 days of the observation period, as indicated by the high number of adults that were recorded during this period. In contrast, the number of *L. paeta* adults only increased slightly, and practically remained unaffected for *L. decolor*.

Regarding the second series of experiments, *L. bostrychophila* was always the dominant competitor. Again, this species was superior in population growth and life table parameters than *L. reticulatus*. For example, *L. reticulatus* egg-to-adult development is slower than that of *L. bostrychophila* [34], [54], [56]. Population growth of *L. bostrychophila* is almost 40 times higher than that of *L. reticulatus* at 30°C [34], [52]. The dominance of *L. bostrychophila* over *L. reticulatus* was manifested regardless of the initial number of *L. bostrychophila* parental females. Hence, even in the vials that contained only one parental *L. bostrychophila* female, *L. reticulatus* became extinct after 140 days. However, the population growth of *L. bostrychophila* was proportional to the initial number of adults, at least during the first 70 days of the observation period. Interestingly, population growth was inversely related to the initial number of parental adults at the last observation period (175 days). We assume that, as noted above, density-dependent mechanisms regulated single-species population dynamics, given that higher numbers of parental females produced higher populations earlier in the observation period, which resulted in more vigorous intraspecific competition and faster food exhaustion. On the other hand, *L. reticulatus* population drastically declined very early in the experiment, when *L. bostrychophila* was present, and this reduction was proportional to the number of parental *L. reticulatus* females. Eventually, *L. reticulatus* was present only when it was alone, which suggests that this species has lower population growth rates and does not colonize as well as *L. bostrychophila*. This species has shorter adult longevity and lower fecundity than *L. bostrychophila*, which clearly stands in agreement with the data of the current study [34], [54]. Throne et al. [57] in a survey in stored wheat in Kansas found that *L. reticulatus* was <1% of the total number of psocid individuals recorded.

We used statistical models with linear and quadratic terms for time as predictors in order to capture U-shaped evolutions of counts. Models with more terms or with time added as a predictor with degree higher than 2 were also explored without adding much to the results. We concluded that coexistence plays a significant role in the population growth and probably on the developmental parameters of psocids, regardless of the final outcome of this competition. Hence, despite that, based on our tests, *L. bostrychophila* was the superior colonizer of the competition, the initial coexistence rate plays an important role in the time that this outcome will occur. In this context, our data documented that coexistence always delays population increase, regardless of the psocid species that coexist. Temperature also plays an important role in this outcome.

To our knowledge, this is the first study in which the inter- and intraspecific competition of stored-product psocid species, expressed as population growth, was assessed, under the basis of limited availability of food or food exhaustion. From the species spectrum tested here, *L. bostrychophila* was clearly the dominant competitor, but the outcome of this competition was based on the other competitor species, temperature, observation period, and initial population. Overall, our tests show that, under conditions of limited food availability, the simultaneous presence of *L. bostrychophila* will lead the populations of *L. decolor* and, especially, *L. reticulatus* to extinction. Conversely, *L. paeta* could coexist with *L. bostrychophila* for a longer period, but in low numbers, which eventually is likely to lead to extinction as well. Further experimentation is needed to assess, apart from population growth, additional factors that are responsible for the superiority of *L. bostrychophila* over the other species tested here, with emphasis on the possible interactions among individuals, such as possible predation activity among individuals or the existence of semiochemical-based population regulators. The results of the present study will contribute by providing the inferences necessary for the development of long-term prediction plans for psocid colonization and extinction patterns in stored grains and related commodities.
